# Complement Evasion by Pathogenic *Leptospira*

**DOI:** 10.3389/fimmu.2016.00623

**Published:** 2016-12-21

**Authors:** Tatiana Rodrigues Fraga, Lourdes Isaac, Angela Silva Barbosa

**Affiliations:** ^1^Laboratory of Complement, Department of Immunology, Institute of Biomedical Sciences, University of São Paulo, São Paulo, Brazil; ^2^Laboratory of Bacteriology, Instituto Butantan, São Paulo, Brazil

**Keywords:** *Leptospira*, leptospirosis, complement system, immune evasion, serum resistance

## Abstract

Leptospirosis is a neglected infectious disease caused by spirochetes from the genus *Leptospira*. Pathogenic microorganisms, notably those which reach the blood circulation such as *Leptospira*, have evolved multiple strategies to escape the host complement system, which is important for innate and acquired immunity. *Leptospira* avoid complement-mediated killing through: (i) recruitment of host complement regulators; (ii) acquisition of host proteases that cleave complement proteins on the bacterial surface; and, (iii) secretion of proteases that inactivate complement proteins in the *Leptospira* surroundings. The recruitment of host soluble complement regulatory proteins includes the acquisition of Factor H (FH) and FH-like-1 (alternative pathway), C4b-binding protein (C4BP) (classical and lectin pathways), and vitronectin (Vn) (terminal pathway). Once bound to the leptospiral surface, FH and C4BP retain cofactor activity of Factor I in the cleavage of C3b and C4b, respectively. Vn acquisition by leptospires may result in terminal pathway inhibition by blocking C9 polymerization. The second evasion mechanism lies in plasminogen (PLG) binding to the leptospiral surface. In the presence of host activators, PLG is converted to enzymatically active plasmin, which is able to degrade C3b, C4b, and C5 at the surface of the pathogen. A third strategy used by leptospires to escape from complement system is the active secretion of proteases. Pathogenic, but not saprophytic leptospires, are able to secrete metalloproteases that cleave C3 (central complement molecule), Factor B (alternative pathway), and C4 and C2 (classical and lectin pathways). The purpose of this review is to fully explore these complement evasion mechanisms, which act together to favor *Leptospira* survival and multiplication in the host.

## Introduction

Spirochetes of the genus *Leptospira* are highly motile Gram-negative bacteria that cause a worldwide zoonosis (1). This bacterium colonizes a wide range of hosts including humans, domestic and wild animal species. Patients with leptospirosis may present either very mild symptoms or subclinical disease (80–90% of infections) or a more severe illness characterized by jaundice, acute renal failure and bleeding (Weil’s disease), or pulmonary hemorrhage syndrome [reviewed in Ref. (2)].

The genus *Leptospira* comprises bacteria having distinct ecological adaptations: exclusively non-pathogenic free-living species, exclusively host-dependent organisms and pathogenic species capable of surviving both inside and outside the host for long periods (3). Molecular phylogenetic analysis of 16S rRNA gene sequences allowed clustering of *Leptospira* species into three groups, comprising pathogens, non-pathogens, and an intermediate group (4).

Upon infection, pathogenic leptospires spread and propagate in susceptible hosts because they have evolved diverse immune evasion strategies. Conversely, saprophytic *Leptospira* strains are highly susceptible to serum bactericidal activity, an observation already made by Johnson and Muschel in the mid-1960s (5). Since our insights into complement evasion mechanisms of *Leptospira* have substantially increased during the last 10 years, we aim here to provide a comprehensive overview of the interactions of this relevant human pathogen with the complement system.

## The Role of the Complement System in the Host’s Defense Against Pathogens

The complement system is composed of more than 50 plasma proteins and receptors. Traditionally considered as one of the first lines of defense against invading microorganisms due to its opsonic, inflammatory, and lytic activities, complement roles extend far beyond pathogen killing [reviewed in Ref. (6)]. Complement effector functions result from activation of three different pathways: classical, alternative, and/or lectin pathways (CP, AP, and LP, respectively). While the AP and LP participate in the innate immunity, the CP is generally activated by the presence of IgG or IgM specifically bound to antigens. The AP is initiated by the spontaneous hydrolysis of an intra-chain thioester bond located in the C3 molecule, while the LP is activated when lectins, such as mannose-binding lectin or ficolins, bind to carbohydrates commonly found on microorganisms’ surfaces. During activation, fragments C3b and C4b are generated and they bind covalently to acceptor surfaces such as immune complexes, foreign, and host cells located on the vicinity of the activation site. On these surfaces, C3 and C5 convertases are formed which further lead to the formation of the membrane attack complex culminating with microorganism lysis. As a consequence of activation, particles opsonized with iC3b, C3b, and C4b are more efficiently internalized by neutrophils, monocytes, and macrophages once bound to complement receptors present on these cells’ membranes. CR2 promotes activation and proliferation of B lymphocytes in the presence of C3d/C3dg fragments covalently bound to antigens inducing the production of antibodies. In addition, C3a and C5a fragments are important anaphylatoxins. They are also chemoattractant factors for inflammatory cells [reviewed in Ref. (7)]. In order to protect the host against self-damage, complement activation is tightly controlled at all stages of the cascade by several soluble and cell surface regulators. C1 inhibitor, Factor I (FI), Factor H (FH), and C4b-binding protein (C4BP) are soluble complement regulators whereas complement receptor type 1 (CR1 or CD35), membrane cofactor protein (MCP or CD46), decay accelerator factor (DAF or CD55), and CD59 are cell-anchored regulatory receptors [reviewed in Ref. (7, 8)].

## Complement Evasion Strategies by *Leptospira*

**Figure 1:**
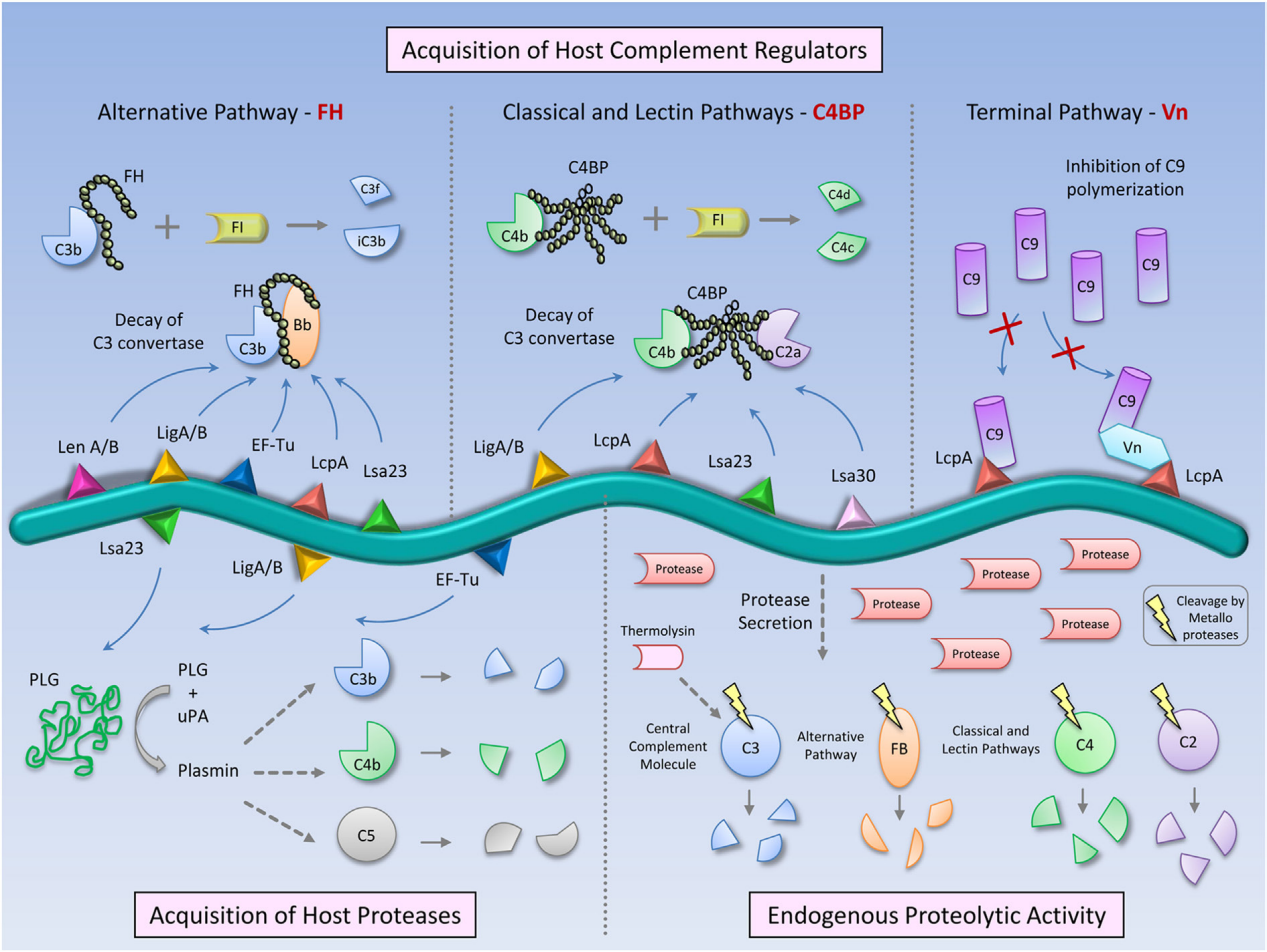
**Complement evasion strategies of pathogenic *Leptospira***. To circumvent the complement system, *Leptospira* has evolved different immune evasion strategies: (i) acquisition of host soluble complement regulators: Factor H (FH)—AP regulator, C4b-binding protein (C4BP) —CP and LP regulator, and vitronectin (Vn) —terminal pathway regulator. FH and C4BP accelerate the decay of the C3 convertases (C3bBb and C4b2a, respectively) and act as cofactors for Factor I in C3b and C4b cleavages. Vn and the leptospiral protein LcpA bind C9 and inhibit its polymerization, thus potentially blocking MAC formation; (ii) acquisition of host proteases: pathogenic *Leptospira* binds plasminogen, which in the presence of activators, such as Urokinase-type plasminogen activator (uPA), is converted in the enzymatically active plasmin. This serine protease cleaves C3b, C4b, and C5, promoting a downregulation of complement activation on the *Leptospira* surface, and (iii) Direct inactivation of complement proteins by *Leptospira* endogenous proteases. Metalloproteases secreted by pathogenic *Leptospira* strains are able to cleave and inactivate the complement proteins: C3 (central complement molecule), Factor B (from AP), and C2 and C4 (CP and LP). Thermolysin is one of the proteases responsible for these cleavages, degrading C3. The combination of host-derived and endogenous factors from pathogenic *Leptospira* enables the bacteria to successfully establish infection and colonize target organs of the host.

Pathogens use a range of strategies to avoid complement attack, and *Leptospira* is no exception to this phenomenon. While pathogenic *Leptospira* strains resist complement-mediated killing, saprophyte *Leptospira* strains are highly susceptible to serum killing (9, 10). Concerning the group of leptospires of intermediate pathogenicity, such as *Leptospira licerasiae*, nothing is known about their response to complement. Pathogenic *Leptospira* escape from complement-mediated killing through: (i) recruitment of host complement regulators; (ii) acquisition of host proteases that cleave complement proteins on the bacterial surface; and (iii) secretion of proteases that inactivate complement in the *Leptospira* surroundings (Figure 1). These mechanisms are universal strategies employed by diverse pathogens including bacteria, viruses, and fungi to circumvent complement attack [reviewed in Ref. (11)].

### Recruitment of Mammalian Host Complement Soluble Regulatory Proteins

Pathogenic *Leptospira* are potentially able to control all pathways of the complement system by acquiring soluble negative host regulators. Control of the AP is achieved by interaction of *Leptospira* with FH, a 155 kDa plasma glycoprotein [443 ± 106 μg/mL in human serum (12)] composed of 20 globular domains (termed complement control protein domains, CCPs). FH inhibits AP activation by preventing binding of Factor B (FB) to C3b, by accelerating the decay of the C3-convertase C3bBb and by acting as a cofactor for the cleavage of C3b by FI (13–15). Serum-resistant *Leptospira* strains bind three members of the FH family: FH itself, Factor H-like protein 1 (FHL-1), and Factor H-related protein 1 (FHR-1) (9, 16). Once bound to the *Leptospira* surface, FH remains functional and promotes FI-mediated cleavage of C3b, thus generating the iC3b fragment (9, 16). Moreover, *Leptospira* survival in FH-depleted serum was shown to be impaired by 60%, and reconstitution of this serum with purified FH up to physiological concentrations restored bacterial survival in a dose-dependent manner, further supporting a role for FH in *Leptospira* serum resistance (16). Control of the CP by pathogenic *Leptospira* is mediated by surface-bound C4BP, a 570 kDa plasma glycoprotein [335 ± 83 μg/ml (12)] that is found in three isoforms with different subunit composition. The major isoform, α7/β1, is a complex of seven α-chains and one β-chain. The other C4BP isoforms in plasma are α7/β0 and α6/β1. Each α-chain is comprised of eight CCPs, and the β-chain is comprised of three CCPs (17). C4BP inhibits CP and LP activation by interfering with the assembly and decay of the C3 convertase (C4bC2a) and by acting as a cofactor for FI in the proteolytic inactivation of C4b (18, 19). Both virulent and culture-attenuated *Leptospira* strains express ligands for C4BP, in contrast to non-pathogenic strains, which have been shown to bind insignificant amounts of this complement regulator (10). As expected, C4b is more efficiently cleaved by FI in the presence of C4BP bound to *Leptospira interrogans* virulent strains, which may probably explain their higher survival rate in normal human serum as compared to culture-attenuated strains (10). Leptospires also acquire vitronectin (Vn) on their surfaces (20). Vn is a glycoprotein that circulates in the bloodstream as a monomer [65–75 kDa, 104 ± 25 μg/mL (12)] or is deposited in the extracellular matrix (ECM) as a multimer that interacts with several macromolecular components, including glycosaminoglycans and collagens (21, 22). Vn plays multiple roles in many biological processes including the regulation of the terminal pathway of complement by inhibiting C5b7 complex formation and C9 polymerization. Once bound to the bacterial surface, it may protect the microorganism against lysis by impairing MAC formation. A number of strains belonging to different *Leptospira* species have been shown to interact with human Vn (20). Acquisition of this terminal pathway regulatory protein may assist *Leptospira* to evade complement attack.

#### *Leptospira* Ligands for Host Complement Regulators

Pathogenic *Leptospira* bind soluble host complement regulators *via* surface proteins and multiple ligands for those regulators have been described. The most extensively characterized complement evasion molecules from *Leptospira* are (i) leptospiral endostatin-like proteins A and B [LenA and LenB (23, 24)], (ii) *Leptospira* immunoglobulin-like (Lig) proteins A and B [LigA and LigB (16, 25)], and (iii) Leptospiral complement regulator-acquiring protein A [LcpA (26)]. All of these proteins have been shown to bind more than one complement regulator and seem to be involved not only in immune evasion but also in adhesion and invasion by interacting with ECM and plasma proteins such as plasminogen (PLG) (27).

*Leptospira interrogans* strains encode up to six distinct paralogous proteins called LenA–F, harboring domains that presumably share structural and functional similarities with mammalian endostatins (24). Two proteins of this family, LenA (formerly called LfhA and Lsa24) and LenB, have affinities for complement regulators. While LenA binds both FH and FHR-1, LenB has been shown to interact only with FH (23, 24).

LigA and LigB are multifunctional proteins capable of interacting with the ECM, cell lines, and complement regulators *in vitro*. The family of Lig proteins is composed of LigA, LigB, and LigC, which respectively consist of 13, 12, and 13 Ig-like domains. In certain *Leptospira* species *ligC* is a pseudogene (28). The *lig* genes are present only in pathogenic *Leptospira* species, and they are expressed during mammalian infection (28). Recombinant LigA and LigB bind FH, FHL-1, FHR-1, and C4BP, thus potentially allowing control of all complement activation pathways (16). FH CCP5 and CCP20 domains interact with both LigA and LigB (16). C4BP CCP4, CCP7, and CCP8 domains are involved in the interaction with both LigA and LigB (29). Fine mapping of the LigA and LigB domains involved in binding to C4BP has demonstrated that interactions occur through the bacterial immunoglobulin-like (Big) domains 7 and 8 (LigA7–8 and LigB7–8) of both LigA and LigB and also through LigB9–10 (29). As FH and C4BP do not compete for binding to Lig proteins, they probably have distinct binding sites on these molecules and may then interact with their targets simultaneously (16). It has been shown that ectopic LigB expression promotes survival of the saprophyte *Leptospira biflexa* in normal human serum (30). LigB binds C3b and C4b directly through repeats 9–11 (LigB9–11) and inhibits both the alternative and classical pathways in hemolytic assays with erythrocytes (30). Given the susceptibility of non-pathogenic *Leptospira* to the alternative pathway (9, 10), the increased resistance of *ligB*-transformed *L. biflexa* to complement killing may be attributed to the acquisition of C3b and FH by these bacteria (30). Further studies extended this observation by demonstrating that expression of both *ligA* and *ligB* genes enhances *L. biflexa*’s resistance to serum killing, as demonstrated by a reduced MAC deposition on *lig-*transformed *L. biflexa* compared to the wild type strain (31).

Pathogenic *Leptospira* species also bind host’s negative complement regulators through a 20-kDa surface-exposed lipoprotein named LcpA. First described as a C4BP-interacting protein (26), LcpA was later shown to bind FH and Vn as well as the terminal pathway component C9 (20). Usually, microorganisms bind FH *via* a common site located inside CCP20 (32). LcpA is no exception to this rule, since a monoclonal antibody directed against CCP20 inhibited binding of FH to LcpA (20). CCP7 and CCP8 domains mediate the interaction of C4BP with LcpA (29). Both FH and C4BP have been shown to remain functional once bound to LcpA, thus being able to act as cofactors for FI (20, 26). LcpA also interferes with the terminal pathway of complement by binding to C9, a molecule that has a key role in MAC formation on bacterial cells. In the presence of LcpA, Zn^2+^-induced C9 polymerization is inhibited *in vitro* and MAC formation on sheep erythrocytes is partially impaired, preventing cell lysis (20). Competitive binding assays indicate that LcpA interacts with C4BP, FH, and Vn through distinct sites (20).

Based on binding affinities, other *Leptospira* proteins have been shown to acquire complement regulators (Table 1). Lsa30 binds C4BP whereas Lsa23 binds both C4BP and FH (33, 34). Interestingly, the moonlighting protein EF-Tu, shown to be surface-exposed in *Leptospira*, also acquires the complement regulator FH (35).

**Table 1:** **Host molecules that interact with *Leptospira* ligands to evade the complement system**.

Host molecule	Ligands (*Leptospira* proteins)	Reference
Factor H (FH)	LenA and LenB (leptospiral endostatin-like proteins A and B)	(24)
	LigA and LigB (*Leptospira* immunoglobulin-like proteins A and B)	(16)
	EF-Tu (elongation factor Tu)	(35)
	LcpA (leptospiral complement regulator-acquiring protein A)	(20)
	Lsa23 (23 kDa adhesin)	(34)
C4b-binding protein	LcpA	(26)
LigA and LigB	(16)
Lsa30 (30 kDa adhesin)	(33)
	Lsa23 (23 kDa adhesin)	(34)
Vitronectin	LcpA	(20)
Plasminogen	EF-Tu	(35)
	Lsa23	(34)
	LigA and LigB	(36)

### Acquisition of Host Proteases That Cleave Complement Proteins on the *Leptospira* Surface

Proteolytic activity is a fundamental tool employed by diverse pathogens to both overcome tissue barriers and evade the immune system (37). Degradation of ECM components favors pathogen spreading and dissemination, while cleavage and inactivation of immune effector molecules dampen the host defense system, allowing an effective establishment of the infection (38, 39).

Pathogenic leptospires circumvent complement attack by the cleavage and inactivation of key complement molecules from the three activation pathways. The degradation of complement proteins may occur indirectly, using host-acquired proteases such as PLG, or directly, by the activity of endogenous proteases produced by pathogenic *Leptospira* strains, as discussed in the next section.

It is well-known that leptospires are able to bind human PLG (40, 41). PLG is a single-chain glycoprotein (92 kDa) that is a key component of the host fibrinolytic system. This proenzyme is found in plasma and extracellular fluids at concentrations of 180–200 µg/mL (42).

Although both saprophytic and pathogenic leptospires bind purified PLG, only pathogenic strains are able to acquire PLG from human plasma (40, 41). The interaction of PLG with *Leptospira* is mediated by bacterial membrane proteins (Table 1), and involves lysine residues, which are probably positioned at the PLG kringle domains. Another interesting finding is that *Leptospira* cell integrity is preserved, since cellular growth is not impaired by PLG binding (40). Once bound to the *Leptospira* surface and in the presence of urokinase-type plasminogen activator (uPA), PLG is converted to enzymatically active plasmin (40, 41). Plasmin is a serine protease that cleaves diverse important biological substrates, including ECM proteins, like fibrinogen, and complement molecules, such as C3b and C5 (43). In this way, pathogenic leptospires coated with plasmin showed reduced deposition of C3b and IgG on their surface, which was probably related to proteolytic degradation of these molecules, potentially reducing opsonization (44). Furthermore, *L. interrogans* serovar Pomona also displayed enhanced survival in human serum when bound to plasmin (44), which reinforces its role in complement resistance.

Several *Leptospira* membrane proteins have been described as PLG ligands (33, 41, 45–56). However, only a few of them were indeed shown to directly interfere with complement activation: the elongation factor Tu [EF-Tu (35)]; LigA and LigB (36) and *Leptospira* 23 kDa surface adhesion [Lsa23 (34)], whose interactions with PLG resulted in the cleavage of C3b, C4b, and/or C5 (Table 1).

### Secretion of Leptospiral Proteases That Directly Inactivate Complement

The *Leptospira* evasion strategies described until know focused on host molecules hijacked by the pathogen to inactivate complement on its surface. Recently, we demonstrated that pathogenic *Leptospira* can produce molecules that are able to directly interfere with the complement system, in a manner independent of the host machinery. It was observed that the culture supernatants of pathogenic, but not of saprophytic *Leptospira* strains, were able to specifically inhibit all the three activation pathways of complement. The inhibitory effect observed could be directly correlated to the proteolytic activity present in these culture supernatants. Indeed, the leptospiral proteases were able to target a wide range of substrates including C3, a key factor in the amplification of the complement cascade, FB from AP, C2, and C4, from CP and LP. These cleavages were observed both with purified complement proteins or normal human serum, which indicates that the leptospiral proteases exert their function in a physiological context and may contribute to bacterial virulence (57).

The proteolytic activity found exclusively in pathogenic *Leptospira* supernatants was almost completely abolished by 1.10-phenanthroline, indicating a major role of metalloproteases in the degradation of complement proteins. A recombinant metalloprotease from the thermolysin family, present only in *Leptospira* pathogenic species, seems to contribute to these cleavages, since it was able to degrade the central complement protein C3 (57).

The degradation and functional inactivation of complement is a key strategy for attenuating diverse immune responses that are dependent on the proper activation of this system (58). The secretion of proteases that directly cleave complement proteins may contribute to *Leptospira* immune evasion, as demonstrated for a wide range of other pathogens (59).

## Concluding Remarks

Complement is a precisely regulated system composed of numerous specific factors that are activated in a cascade-like manner. This multifactorial cascade nature provides diverse targets for possible interferences by pathogen-derived evasion molecules (58). Most successful human pathogens have developed multiple parallel mechanisms of evading the complement system (60). *Leptospira*, which is a highly invasive spirochete, is a good example of a pathogen that employs diverse strategies to circumvent complement activation (Figure 1). The combination of host-derived and endogenous factors enables these spirochetes to successfully establish the infection and colonize target organs of the host. Therefore, *Leptospira* ligands of host regulators and secreted proteases constitute potential sites for immune interference, either as vaccine candidates or as targets for therapeutic agents in the development of new treatments and prophylactic approaches in leptospirosis.

## Author Contributions

TF participated in the drafting of the article and prepared the tables and figures. LI performed a critical revision and approved the final version to be published. AB participated in the drafting of the article and performed a critical revision of the final version.

## Conflict of Interest Statement

The authors declare that the research was conducted in the absence of any commercial or financial relationships that could be construed as a potential conflict of interest.
